# ‘None of my ancestors ever discussed this disease before!’ How disease information shapes adaptive capacity of marginalised rural populations in India

**DOI:** 10.1371/journal.pntd.0009265

**Published:** 2021-03-11

**Authors:** Festus A. Asaaga, Mujeeb Rahman, Suresh D. Kalegowda, Jagadeesh Mathapati, Irfanahemad Savanur, Prashanth N. Srinivas, Tanya Seshadri, Darshan Narayanswamy, Shivani K. Kiran, Meera A. Oommen, Juliette C. Young, Bethan V. Purse

**Affiliations:** 1 UK Centre for Ecology & Hydrology, Wallingford, United Kingdom; 2 Ashoka Trust for Research in Ecology and the Environment, Bengaluru, India; 3 ICAR-National Institute of Veterinary Epidemiology and Disease Informatics, Bengaluru, India; 4 Institute of Public Health, Bangalore, India; 5 Department of Health and Family Welfare Services, Government of Karnataka, Shivamogga, India; 6 ICMR-National Institute for Traditional Medicine, Belgavi, India; 7 UK Centre for Ecology & Hydrology, Edinburgh, United Kingdom; 8 Agroécologie, AgroSup Dijon, INRAE, Univ. Bourgogne, Univ. Bourgogne Franche-Comté, Dijon, France; Institute of Continuing Medical Education of Ioannina, GREECE

## Abstract

Smallholder farmer and tribal communities are often characterised as marginalised and highly vulnerable to emerging zoonotic diseases due to their relatively poor access to healthcare, worse-off health outcomes, proximity to sources of disease risks, and their social and livelihood organisation. Yet, access to relevant and timely disease information that could strengthen their adaptive capacity remain challenging and poorly characterised in the empirical literature. This paper addresses this gap by exploring the role of disease information in shaping the adaptive capacity of smallholder farmer and tribal groups to Kyasanur Forest Disease (KFD), a tick-borne viral haemorrhagic fever. We carried out household surveys (n = 229) and in-depth interviews (n = 25) in two affected districts–Shimoga and Wayanad–in the Western Ghats region.

Our findings suggest that, despite the generally limited awareness about KFD, access to disease information improved households’ propensity to implement adaptation strategies relative to households that had no access to it. Of the variety of adaptation strategies implemented, vaccination, avoiding forest visits, wearing of protective clothing and footwear, application of dimethyl phthalate (DMP) oil and income diversification were identified by respondents as important adaptive measures during the outbreak seasons. Even so, we identified significant differences between individuals in exposure to disease information and its contribution to substantive adaptive action. Households reported several barriers to implement adaptation strategies including, lack of disease information, low efficacy of existing vaccine, distrust, religio-cultural sentiments, and livelihood concerns. We also found that informal information sharing presented a promising avenue from a health extension perspective albeit with trade-offs with potential distortion of the messages through misinformation and/or reporting bias. Altogether, our findings stress the importance of contextualising disease information and implementing interventions in a participatory way that sufficiently addresses the social determinants of health in order to bolster households’ adaptive capacity to KFD and other neglected endemic zoonoses.

## Author summary

There is widespread consensus that smallholder farmer and tribal communities are highly vulnerable to zoonotic diseases (illness passed between animals and people). This is largely due to their relatively poor access to healthcare and closeness to sources of disease risks. Although access to relevant and timely disease information could enhance their adaptive capacity, remain difficult and poorly understood. Drawing on survey data (n = 229) and in-depth interviews (n = 25) in two affected districts (Shimoga and Wayanad); we explored the role of disease information in shaping adaptation to Kyasanur Forest Disease (KFD), a tick-borne viral haemorrhagic fever. We found that despite the limited awareness about KFD, access to disease information improved households’ likelihood to implement adaptation strategies as compared to households that had no access to it. Households identified several barriers to implementation of adaptation strategies including, lack of disease information, low efficacy of existing vaccine, distrust, religio-cultural sentiments, and livelihood concerns. Our findings highlight the relevance of contextualising disease information and implementing interventions in a participatory manner in order to strengthen households’ adaptive capacity to KFD and other neglected endemic zoonoses.

## Introduction

Neglected zoonotic diseases continue to have increasingly profound impacts on the health, social and livelihood organisation of many in low-middle-income countries (LMICs), particularly for marginalised rural populations who constitute the most vulnerable of vulnerable groups [1–4]. As the burdens of these emerging diseases continue to evolve, exacerbated by wide-ranging socio-political, ecological and environmental change drivers [5,6], marginalised and vulnerable groups need some degree of adaptive capacity, and ability to utilise existing resources and leverage potential opportunities to alleviate or minimise potential damage or consequences of disease hazards [3,7]. This is vitally important considering that the recent zoonoses research forecasts that environmental changes and other socio-ecological drivers are likely to exacerbate the existing disease burdens, particularly in LMICs (including India) with relatively weak health systems [1,7]. The Intergovernmental Panel on Climate Change (IPCC) for instance, in its Fifth Assessment Report, predicted with high confidence that climate change, among other stresses, will reduce agricultural yields, aggravate water stress and alter the ecology of infectious disease vectors across the Global South through changes in temperature and precipitation patterns [8,3,9]. Nonetheless, the ability of small-holder farmer and tribal communities’ to adapt may be impaired by inequities that systematically constrain access to essential and productive resources, such as reliable disease information [10,11,12]. Disease information in the context of this study refers to the various set of information and messages that are relevant for the prevention and control of KFD such as adopting personal protection measures, use of tick repellents (e.g. dimethyl phthalate (DMP) oil, diethyltoluamide (DEET)) prior to visiting forest, vaccination of people in disease-prone areas and other risk-prone activities. Although the importance of meaningful community engagement in public health interventions has been recognised [13], to date, there is limited empirical examination of the role of disease information in shaping the adaptive capacity of vulnerable groups to emerging zoonotic disease risks [14]. The complex and changing disease dynamics (shaped by the ecology and evolutionary biology of infectious disease vectors and hosts [1,15,6] has meant that access to up-to-date information remains crucial to successful adaptation of communities [16]. We thus address this knowledge gap by exploring how the delivery and access to disease information influences the adaptive capacity of small-holder farmer and tribal communities to emerging zoonotic disease risks, focussing on the context of India. India provides an excellent setting to examine the burden and impacts of neglected zoonotic diseases such as KFD, rabies and scrub typhus considering: (1) its status as one of the four global zoonoses ‘hotspots’ [4], and (2) the millions of marginalised rural populations whose livelihood security, health and welfare are increasingly under threat from several emerging and re-emerging zoonotic disease risks [1,17,4,18]. Given the contextual similarities, this study could potentially be useful for other LMICs settings as it provides broad-level empirical insights on the interplay between disease information dynamics and adaptation planning.

Vulnerability in this context is understood as the degree to which individuals and/or groups are susceptible to the adverse effects (both direct and indirect) of zoonotic disease and its associated stressors. Within this context, we focus on the Kyasanur Forest Disease Virus (KFDV), a tick-borne virus causing debilitating and potentially fatal haemorrhagic disease in people in the Western Ghats region of south India. Available statistics indicate that KFDV affects approximately 500 persons annually [19,20], with the first case reported in the Kyasanur forest in Soraba taluk of Shimoga district in Karnataka state of southern India [21]. Although the KFDV has been known to be prevalent locally for several decades (since its description in late 1950s), there is currently no specific treatment for the disease [22]. In recent times, the geographical range of KFDV has significantly expanded from the Shimoga district in Karnataka to adjacent states to the north, west and south of Shimoga along the Western Ghats, including Kerala, Goa, Maharashtra and Tamil Nadu [19,22–26]. The increasing geographical spread of the KFDV has culminated in its recent recognition (in 2019) as a disease of national public concern [27].

Although KFDV has been on the rise since 2005 [23], more recent outbreaks (i.e. from 2012 onwards) have been particularly devastating. Available statistics indicate that about 1929 human KFD cases were reported between 2010 and 2019, with 656 (representing 65%) reported in Shimoga district in Karnataka alone [1]. Though humans are not involved in onward transmission, being “dead-end” hosts, the suspected low efficacy of existing vaccines and the lack of specific treatment for KFD mean that disease adaptation is still critical. At the same time, while existing control efforts have somewhat minimised spread through vaccination campaigns, the overall effectiveness of such interventions have been considerably undermined by poor vaccination coverage, shortage of vaccines and exclusion of certain eligible populations [22,28,29]. Kiran et al. [28] for instance, reported that the success of the December 2013-April 2014 outbreak and vaccination strategy in the Shimoga district was marred by low vaccine coverage and the unanticipated spread of KFDV to newer unvaccinated areas (>5 km away from villages vaccinated in the previous year). Likewise, the study by Oliveira et al. [22] on vaccination coverage in KFD-affected villages in Goa state reported that some key groups of the eligible population were left out of the vaccination campaigns. As expected Oliveira et al. concluded that the limited vaccine efficacy has meant that multiple doses (two initial dose and a booster) are required to achieve full protection. With recent KFD spread and modelling studies forecasting potential future outbreaks in previously unaffected areas in India [1], the need for relevant disease information to inform local-level coping or adaptation planning and decision-making become even more imperative [27].

While zoonoses vulnerability and adaptation is gaining widespread policy attention amidst the global ‘One Health’ movement and more recently the COVID-19 pandemic, public health policymakers and planners at the national and international levels are grappling with feasible pathways to bolster the adaptive capacity of marginalised rural populations to emerging disease threats [30,13,31]. In the Indian context, despite progress made in the containment of several zoonotic infections [17], existing interventions have been criticised on the grounds that they are largely focussed on the biomedical and/ or technical strategies (including vaccination, treatment) overlooking important socio-economic and cultural aspects that affect sensitivity and exposure to disease risks [32–34]. Since disease causation, spread and control are multi-layered with socio-economic, cultural and political underpinnings, responses must be rooted in *inter alia*, the socio-cultural practices and processes of populations responding to disease risks [13,33,35]. While a number of disease adaptation initiatives (including vaccination, awareness campaigns, use of tick repellents, etc.) are being promoted in affected areas by health authorities and other non-governmental support groups to enable local populations cope effectively and successfully adapt [27], the uptake of these measures varies within and across groups. It therefore follows that enhancing the adaptive capacity of smallholder farmer and tribal communities is predicated on understanding their current preparedness and adaptive capacity to inform on-going and future interventions. Nevertheless, the often-homogenous characterisation of small-holder and tribal groups has meant masking critical and nuanced differences that may exist in terms of their differentiated vulnerabilities and how they are exposed to KFD infection, as this paper elucidates. Aside from disease vulnerabilities, existing inequalities (driven by the interplay of socio-economic and political forces) also operate to further exacerbate their vulnerabilities and health outcomes. Against this background, this paper explores how small-holder farmer and tribal households managed the impacts of past KFD outbreaks. By focussing on past KFD outbreaks, it affords the opportunity to understand the actual and potential adaptive capacity of the sampled focal communities with respect to their local response.

Drawing inspiration from Maguire-Rajpaul et al.’s [10] work on the impact of agricultural information on smallholder farmers’ adaptive capacity, we demonstrate how access to and utilisation of disease information affects the adaptive capacity of vulnerable groups in affected villages in the Shimoga and Wayanad districts of south India. We ask the overarching question: *“how and to what extent does access to and utilisation of disease information shape/affect/influence small-holder and tribal groups’ adaptation to KFD*?*”* The following sub-questions guided the investigation:

- What are the existing sources of disease information available to households?
- What are the key adaptive actions implemented by them; and how are these actions supported (or not) by the access to relevant disease information?
- How and to what extent is access to disease information important in shaping small-holder farmer and tribal groups’ adaptive actions?

### Conceptualising disease information and adaptive capacity nexus

**Fig 1:**
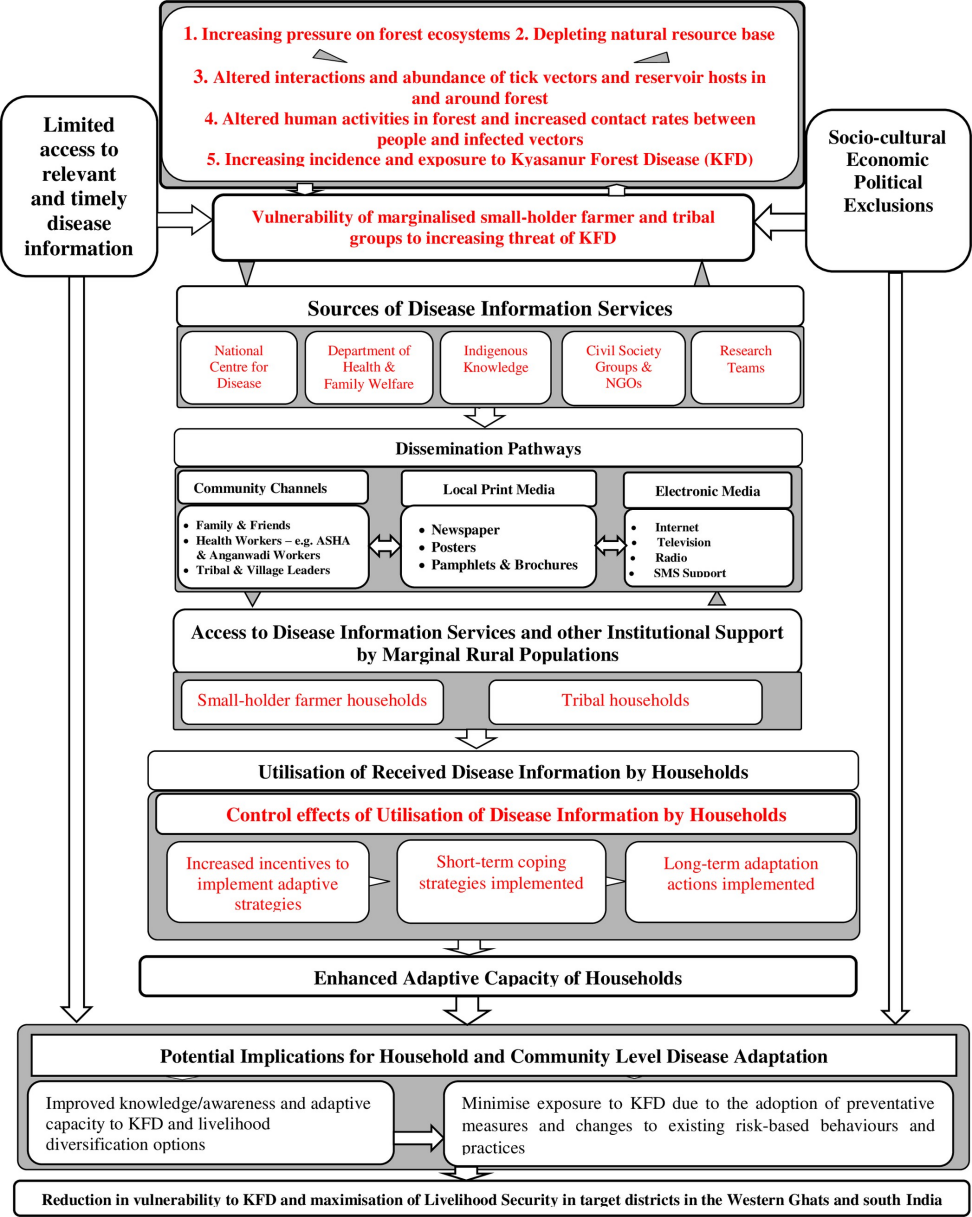
Conceptual Framework of the study. Source: Authors’ modification based on Asaaga [36].

Access to disease information and support services play a critical role in building adaptive capacity and preparedness in the face of emerging disease risks [34,33]. The conceptual model as shown in Fig 1 is premised on the postulation that vulnerable groups effectively adapt if they have access to relevant and timely disease information, which in turn bolsters their resilience to disease risks. It therefore follows that an individual farmer or household’s capacity to implement adaptive strategies based on disease information would be influenced by the interplay of household, community and institutional factors.

As illustrated in Fig 1, the increasing anthropogenic pressure exemplified by the progressive alteration of forest ecosystems has heightened the risk of exposure of humans and livestock to multi-host zoonotic pathogens in the Western Ghats region. This has particularly meant increased susceptibility of forest-dependent communities to KFD by virtue of their resource-based livelihoods, remoteness from healthcare infrastructure and other socio-economic inequalities [1]. Within this paradigm, improving access to relevant and timely disease information and support services is seen as a plausible pathway that provides the basis for bolstering the adaptive capacity of marginalised groups by incentivising their individual and collective agency to implement adaptive strategies to minimise or avoid exposure to KFD whilst maximising the livelihood benefits they derive from forests.

Within this context, a considerable body of literature has characterised the burden and impacts of emerging zoonoses in LMICs, particularly among the poor and marginalised groups [34,30]. Central to the theoretical and policy debates on neglected zoonotic infections is the identification and operationalisation of response mechanisms that reduce vulnerability and enhance local adaptive capacity. In effect, the summation of the different response mechanisms utilised to cope and/ or adapt to disease risks constitute disease adaptation. Synonymous to vulnerability, adaptation has been defined variously in the scholarly literature largely reflecting the theoretical or disciplinary orientation of proponents. The IPCC [8] defines adaptation as “adjustments made in natural or human systems in response to actual or expected disease risk and its impacts, which minimises harm or exploits beneficial opportunities”. Embedded in this broad definition are two important inter-related concepts viz. coping and adaptation, that determine overall adaptive capacity of a target population. Whereas coping is generally described as the short-term mechanisms to ensure survival, adaptation on the other hand, is akin to the long-term shifts in behaviour and practices which reduces vulnerability [37]. In this regard, short-term adaptive strategies are often conditioned to help improve survival within the scope of existing responses and value systems, while long-term responses involve a fundamental shift in the social and livelihood patterns in order to be considered transformative [37]. For instance, a tribal household may in the long-term modify its honey-extraction practices in order to reduce risk of exposure to KFD. In any event, determining whether to implement short or long-term adaptive strategies is largely dependent on the contextual situation (including, socio-cultural, economic and political realities) [37,38].

Different individuals or social groups may have different capacities to act on received disease information in their decision-making related to livelihood adaptation. Other observers have challenged the conceptual distinction between coping and adaptive strategies positing that the definition of former is not definitively different from the latter [39]. In other words, the distinction between the coping and adaptive strategies may be superficial as short-term coping strategies could become adaptation actions in the long-term [39,40]. Aside from the coping-adaptation dichotomy, a critical scholarship has also theorised adaptation responses as either autonomous or planned based on pre-existing local knowledge and/ or long-term deliberate planning implemented over time [37,39,41]. The ‘autonomous’ adaptation framing implicitly suggests that adaptive responses are independently initiated and/or self-governed devoid of any external control, which neglects the people-social environment-ecosystem interdependence on both spatial and temporal scales, and the lack of control that many otherwise marginalised groups have over drivers of change [37].

Focussing on neglected zoonoses, several commentators have identified different ‘bottom-up’ coping and/ or adaptation strategies employed by individuals and groups in responding to different zoonotic disease risks [34]. Of the myriad of strategies, household socio-economic status, knowledge, personal networks, diversification of farm and non-farm activities amongst others have been theorised as influencing local adaptive capacity to emerging zoonotic disease risks [33,13]. Other scholars have also observed that the capacity to adapt is predicated on the socio-economic and political power structures (within which disease risks manifest) as they operate to either enhance or constrain access to productive resources, including access to relevant disease information [30,13]. Against this backdrop, this study focusses on how access to and utilisation of disease information by small-holder farmer and tribal groups could bolster their adaptive capacity in two case study areas. Of particular interest is whether access to and utilisation of disease information of marginalised rural populations underpins their adaptation decision-making. In this regard, we hypothesize that disease information is critical to bolstering the adaptive capacity of small-holder farmer and tribal households’ and seek to refine the understanding of how disease information could build overall resilience to emerging and endemic zoonotic disease risks.

## Research design and methodology

### Ethics statement

Participation in this research was voluntary and all participants gave their full prior-informed verbal and written consent before the conduct of the interviews which lasted between 45 minutes to an hour. The collated data from the survey and interviews were duly anonymised using de-identifiers or pseudonyms (e.g. SA1, WD1) to safeguard the confidentiality of participants. The study protocol was approved by the Research Ethics Committees at the Ashoka Trust for Research in Ecology and the Environment (IRB/CBC/0003/ATV/07/2018), the Institute of Public Health Bangalore (IEC-FR/04/2017) in India, and received a Favourable Ethical Opinion from the Liverpool School of Tropical Medicine Research Ethics Committee in the United Kingdom (research protocol 17–062).

### Selection and description of study sites

**Fig 2:**
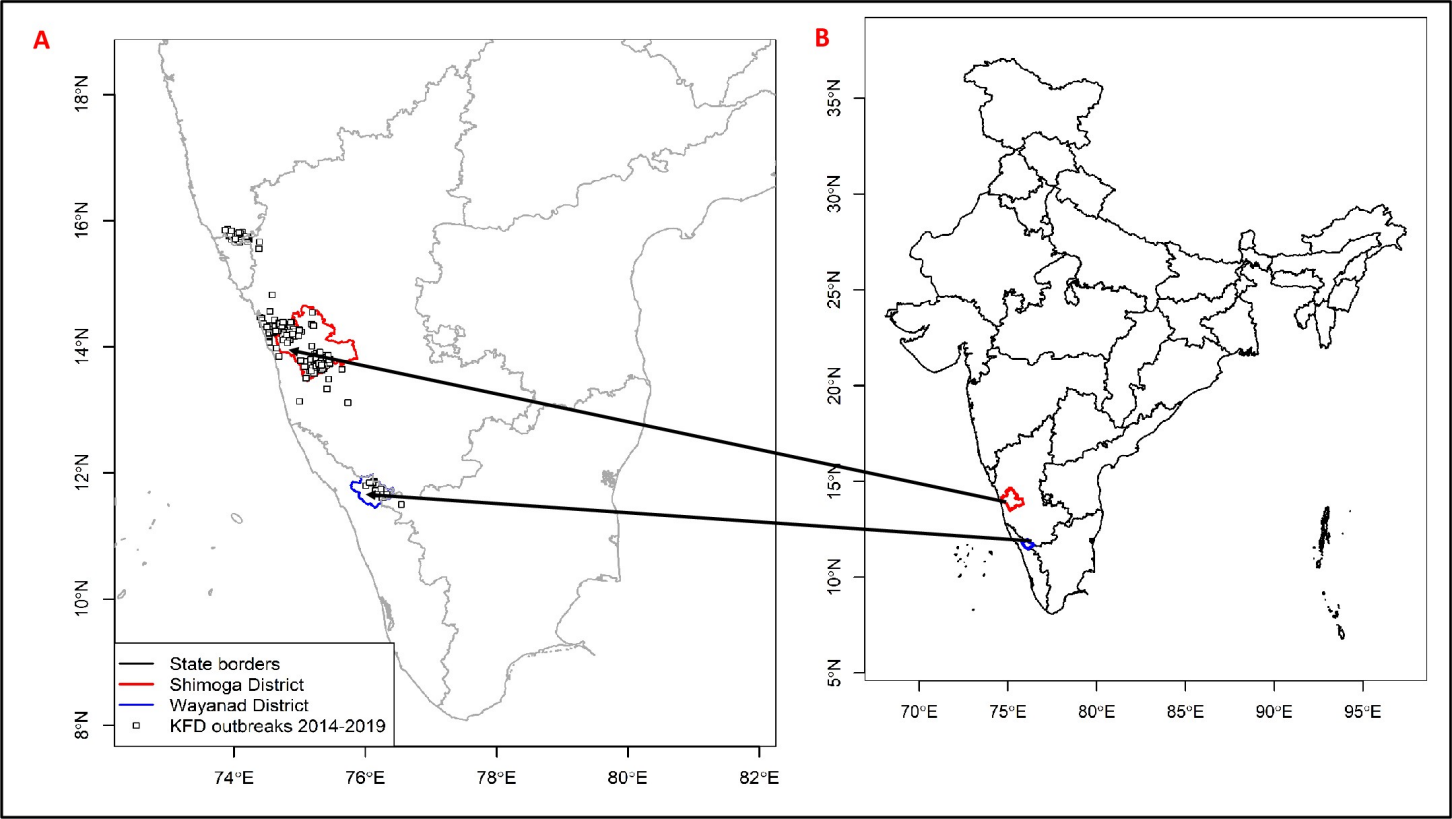
An overview map of Shimoga and Wayanad districts in regional and national contexts. The administrative boundary dataset used in this figure is from HindudstanTimesLabs (https://github.com/HindustanTimesLabs/shapefiles/), reproduced under the MIT License. Human case data are from the Department of Health and Family Welfare Services, Government of Karnataka.

This paper is part of an interdisciplinary research study, ***MonkeyFeverRisk*** [42] that–among other objectives—sought to develop innovative inter-disciplinary frameworks to help rural forest-based communities limit exposure to zoonotic diseases whilst maximising the livelihood benefits they derive from tropical forests in south India. The study was conducted in two districts, Shimoga and Wayanad, situated in Karnataka and Kerala states respectively along south India’s Western Ghats region (Fig 2). Shimoga and Wayanad cover an area of 8,465 km^2^ and 2,131 km^2^, with an estimated population of 1,752,753 and 8,017,420 respectively [43]. Like other parts of the Western Ghats, the forests of Shimoga and Wayanad have been degraded and fragmented through decades of timber extraction, industrial development and increases in agriculture and plantations [44,45]. The choice of the two study regions was informed by a number of theoretical and empirical postulations. First, Shimoga and Wayanad districts are considered susceptible to KFD outbreaks given their respective histories as long-affected (having outbreaks since KFD discovery in March 1957) and recently affected areas (since 2014). Second, the existence of considerably higher number of households listed to the Scheduled Castes and Tribes (*dalit* and *adivasi* populations–collective terms for the diverse socio-economically and culturally distinct indigenous populations of India), whose livelihood organisation is primarily linked to forest resources positioned the study areas as suitable settings for this investigation. Together, the selection of the two contrasting contexts (i.e. based on differences in politico-administrative structures, socio-economic characteristics as well as KFD endemicity) afforded the unique opportunity to explore important contextual differences that may exist in terms of disease information dynamics and adaptation planning and implementation. For instance, the long history of KFD vis-à-vis the several (past and ongoing) interventions in Shimoga enabled a detailed appreciation of how the transmission of disease information has shaped the local communities’ coping and adaptation to KFD and its associated stressors overtime.

## Methods

The study adopts a mixed methods approach combining quantitative and qualitative techniques (including household surveys, key informant interviews and personal observations) to collate and analyse empirical data on the dynamics of disease information and its impact on KFD adaptation. A multistage sampling approach was employed in the selection of study participants. In consultation with local stakeholders, the initial stage was the purposive selection of the two districts–Shimoga and Wayanad–based on pre-generated list of KFD affected districts along the Western Ghats as elucidated above (see Fig 2). This was followed by the selection of 30 villages (from the pre-defined list of affected and unaffected villages) based on stratification by forest proximity. In each of the surveyed villages, six household heads were randomly selected and interviewed (depending on availability and willingness of the household heads to participate in the survey). The household survey comprised of 229 small-holder and tribal households from Shimoga (n = 157) and Wayanad (n = 72) conducted in August 2019. A team of four research assistants (with a good working knowledge of the surveyed villages) were trained; who in turn piloted and administered the survey instrument. The research assistants were all natives from the two focal districts and had had previous experience of data collection in the study areas. Moreover, the research assistants’ good understanding of cultural norms and practices in the surveyed communities proved useful in negotiating access both for the household survey and subsequent key informant interviews. The questionnaire instrument elicited participants’ views and experiences with respect to KFD and its related impacts on livelihoods, application of a range of adaptation strategies and challenges they faced in implementing these strategies. The survey data were descriptively and inferentially analysed using the Statistical Package for Social Science (SPSS version 20) and reported in tables and charts. Pearson Chi-square tests were performed to detect significant differences (p<0.05) between groups based on study region, gender, age and caste. A knowledge summation score (null, low, medium, high) was developed based on five items assessed (for a maximum of 5 positive responses as a function of participants’ awareness of KFD): (null (0 = no positive or ‘yes’ answer), low (1 = 1 positive answer), medium (2 = 2 or 3 positive answers) and high (3 = 4 or 5 positive answers)). Participants who declared that they had never heard about KFD before the survey were automatically given 0 for this score [46].

The quantitative data was supplemented with a series of key informant interviews (n = 25) conducted (between August 2019 and March 2020) with local stakeholders (directly or indirectly) affected by KFD management (including KFD survivors, village elders and tribal chiefs, district and taluka disease managers and other healthcare workers etc.) in the surveyed communities. The selection of participants was based on a purposive and snowball sampling process, with interviews mainly conducted in *Kannada* and *Malayalam*, the predominant local languages spoken in the surveyed communities in Shimoga and Wayanad respectively. The interviews were on one-to-one basis mostly in the homes of participants except for a few participants who were contacted at their offices, according to their preference. On average, the interviews lasted between 45 minutes and 1 hour and were audio-tapped with the prior consent of participants. The careful selection of the interviewers (based on nativity and local language proficiency) afforded the ease of accessing appropriate key informants, validating and obtaining additional grounded information, particularly on disease outbreak histories, livelihood and occupational dynamics. The sample size was determined by data saturation–i.e. the point where no new themes from participants’ experiences emerged. Considering the localised nature of KFD outbreaks and the sample homogeneity, data saturation was deemed to have been reached during the data collection when no new information was obtained relative to the study objectives. The interviews mainly revolved around participants’ views and lived experiences with KFD, their coping and adaptation strategies etc. which was transcribed and analysed following a thematic and content analysis approach as described by [47]. We read the interview transcripts (and field notes) repeatedly to gain an in-depth understanding of the meanings conveyed, identifying important phrases, formulating and validating meanings, identifying and organising patterns and contradictions into emergent themes through an open coding process. Identified emergent themes were further organised into clusters and categories after which full description of themes was undertaken. The developed themes were subsequently analysed and triangulated with the survey data and other relevant secondary information collated based on which inferences and conclusions were drawn [48,49]. S1 Table outlines the main themes identified through the interviews and S2 Table identifies key informants interviewed (see supplementary data file).

## Results

### Demographic Information and Dynamics of KFD-related Awareness

Over two-thirds of the sampled respondents interviewed (68.6%) were male largely, which is reflective of the social and occupational context (Table 1). Nearly 75% respondents are of working age (i.e. 15–64 years cluster) with mean ages of 57.6 and 47.1 in Shimoga and Wayanad respectively. On the social stratification scale, the overwhelming majority of respondents (84.2%) reported belonging to a lower caste (i.e. scheduled tribe, scheduled caste and other backward classes (OBC) as per statutory classification of caste groups in India). Of the 157 respondents interviewed in Shimoga, about 96% identified themselves as belonging to a lower caste relative to the 58.3% in Wayanad. A majority of respondents (70.3%) reported some form of formal schooling, with a relatively even distribution across the two study areas. In Shimoga for instance, whereas 30.6% of respondents claimed they had no formal education, 26.5% attained secondary level and 7% tertiary education respectively. In Wayanad, 28% had no formal education, 30% completed secondary level and 4% tertiary education respectively.

**Table 1:** The demographic characteristics of respondents by study region.

Socioeconomic characteristics	Study sites		Full sample (N = 229)
	Shimoga (N = 157)	Wayanad (N = 72)	
**Gender Household Head**			
Female	39 (24.8%)	33 (45.8%)	72 (31.4%)
Male	118 (75.2%)	39 (54.2%)	157 (68.6%)
**Age Classification**			
Aged 15–24 years	1 (0.6%)	1 (1.4%)	2 (0.9%)
Aged 25–64 years	102 (65%)	67 (93.1%)	169 (73.8%)
Aged > 65 years (the elderly)	54 (34.4%)	4 (5.6%)	58 (25.3%)
**Social Stratification (Caste)**			
Scheduled caste	8 (5.1%)	3 (4.2%)	11 (4.8%)
Scheduled tribe	26 (16.5%)	22 (30.5%)	48 (20.9%)
Other backward caste	117 (74.5%)^b^	17 (23.6%)^b^	134 (58.5%)
Other (Upper caste)	6 (3.8%)	30 (41.7%)	36 (15.7%)
**Educational Level**			
No formal education	48 (30.6%)	20 (27.8%)	68 (29.7%)
Completed primary level education	36 (22.9%)	20 (27.8%)	56 (24.5%)
Completed middle level education	20 (12.7%)	5 (6.9%)	25 (10.9%)
Completed matriculation/secondary level	33 (21.0%)	12 (16.7%)	45 (19.7%)
Higher secondary/ Pre-university	9 (5.7%)	10 (13.9%)	19 (8.3%)
Technical diploma		2 (2.8%)	2 (0.9%)
Tertiary education	11 (7.0%)	3 (4.2%)	14 (6.1%)
**Religious Affiliation**			
Hindu	148 (94.3%)	51 (70.8%)	199 (86.9%)
Christian	1 (0.6%)	11 (15.3%)	12 (5.2%)
Muslim	8 (5.1%)	10 (13.9%)	18 (7.9%)
**Poverty Status**			
Below poverty line (BPL)	139 (88.5%)	46 (63.9%)	185 (80.8%)
Above poverty line (APL)	18 (11.5%)	26 (36.1%)	44 (19.2%)
**Primary Occupation**			
Agriculture-based	106 (67.5%)	24 (33.3%)	130 (56.8%)
Non-agriculture based*	15 (9.6%)^b^	35 (48.6%)^b^	50 (21.8%)
Unemployed	36 (23%)	13 (18.1%)	49 (21.4%)

^a^ Significant at p ≤ 0.01

^b^ Significant at p ≤ 0.05

The fairly low educational attainment (8% of total sample completed higher secondary) in the surveyed communities across the two study regions could have negative implications, in terms of disease literacy by constraining access to formal sources of disease information such as newspapers, internet and information leaflets. Concerning income status, the overall results suggest that a significant proportion of surveyed households in Shimoga (88.5%) and Wayanad (63.9%) were classed as living ‘below poverty line’ [see 50], implying that they earned less than Rs. 32 (£0.35) a day, as per the revised Government of India poverty index [43]. Within this context, agriculture constituted the primary source of employment, with over half of surveyed households (56.8%) engaged in agriculture. Further disaggregation by study area revealed significant regional differences in terms of non-agriculture based employment, as nearly half (48.6%) of surveyed households in Wayanad were engaged in other primary livelihood activities as against just 9.6% of households in Shimoga within the same category (χ^2^ value 44.12, p≤ 0.05). This highlights relative regional differences in socio-economic positions with significant implications for zoonotic disease vulnerability, local disease control strategies and pathways to adaptation available to individuals and households.

### Dynamics of awareness and perceptions relating to KFD

To afford a better understanding of the disease information and adaptive capacity nexus in the study areas, it was pertinent to assess respondents’ level of awareness and perceptions about KFD. As shown in Table 2, almost three-quarters of all respondents (n = 166) confirmed their awareness of KFD when asked whether they had heard of the disease prior to the survey, with 26.6% reporting them and/or know someone who had contracted the disease.

**Table 2:** Awareness and perceptions about KFD by study area.

Categories	Study Regions		Full sample (N = 229)
	Shimoga (n = 157)	Wayanad (n = 72)	
**Past history with KFD**			
Have ever had/contracted KFD	10 (6.4%)	7 (9.7%)	17 (7.4%)
Know someone with KFD	28 (17.8%)	16 (22.2%)	44 (19.2%)
Heard of KFD/ Monkey fever	119 (75.8%)	47 (65.3%)	166 (72.5%)
Vaccinated against KFD	93 (59.2%)	14 (19.4%)	107 (46.7%)
**Awareness of KFD**			
Exposure (awareness of the likelihood of people to contract KFD)	86 (54.8%)	29 (40.3%)	115 (50.2%)
Risk zone (awareness of KFD linked to forest usage)	112 (71.3%)	5 (6.9%)	117 (51.1%)
Transmission mode (know that KFD is transmitted by an infected tick bite)	57 (36.3%)	35 (48.6%)	92 (40.2%)
Coping (know what measures to undertake in the event of suspected KFD infection)	36 (22.9%)	10 (13.9%)	46 (20.1%)
Prevention (awareness of any prevention measures, including vaccination)	96 (61.1%)	15 (20.8%)	111 (48.5%)
**Aggregated score on level of awareness**			
High (% with aggregate score of 4 or 5)	44 (28.0%)^b^	5 (6.9%)^b^	49 (21.4%)
Medium (% with aggregate score of 2 or 3)	49 (31.2%)	13 (18.1%)	62 (27.1%)
Low (% with aggregate score of 1)	20 (12.7%)	15 (20.8%)	35 (15.3%)
Null (% with aggregate score of 0)	44 (28.0%)^b^	39 (54.2%)^b^	83 (36.2%)
**Perception about KFD**			
High perceived severity of KFD	89 (56.7%)	2 (2.8%)	91 (39.7%)
KFD as a major/ significant health issue in the region	110 (70.1%)	49 (68.1%)	159 (69.4%)
KFD not at all significant health issue in the region	47 (29.9%)	23 (31.9%)	70 (30.6%)
People are likely to contract KFD	86 (54.8%)	29 (40.3%)	115 (50.2%)
Extremely worried about contracting KFD	30 (19.1%)	7 (9.7%)	37 (16.2%)
Worried about contracting KFD	35 (22.3%)	29 (40.3%)	64 (27.9%)
Not worried about contracting KFD	92 (58.6%)	36 (50.0%)	128 (55.9%)

^a^ Significant at p ≤ 0.01

^b^ Significant at p ≤ 0.05

Although most households (totalling 69%) generally reported that KFD constituted a significant health concern in their respective villages, there were marked differences in terms of the perceived level of severity and whether people are likely to contract the disease. In this regard, the overall results indicate that households have a diverging perception about KFD, ranging from worried and extremely worried (44%) to not at all worried about the disease (56%). Despite half of the households in Wayanad expressing worry about KFD, an even lesser proportion (40.3%) perceived the likelihood of people contracting it. By contrast, the Shimoga data indicate that despite a majority of households (58.6%) not being worried about KFD, a fairly high percentage of respondents (54.8%, n = 86) expressed the likelihood of people contracting it. A further disaggregated analysis revealed statistically significant difference between prior experience of KFD (contracting or knowing someone with KFD) and feeling worried about contracting same, particularly in Shimoga. For example, the overall results indicate that as many respondents who had prior experience with KFD in Shimoga (63.2%, 24/38) also reported being worried about contracting it (χ^2^ value 8.02, p≤ 0.05). A similar pattern is portrayed in the Wayanad data but is not statistically significant.

**Fig 3:**
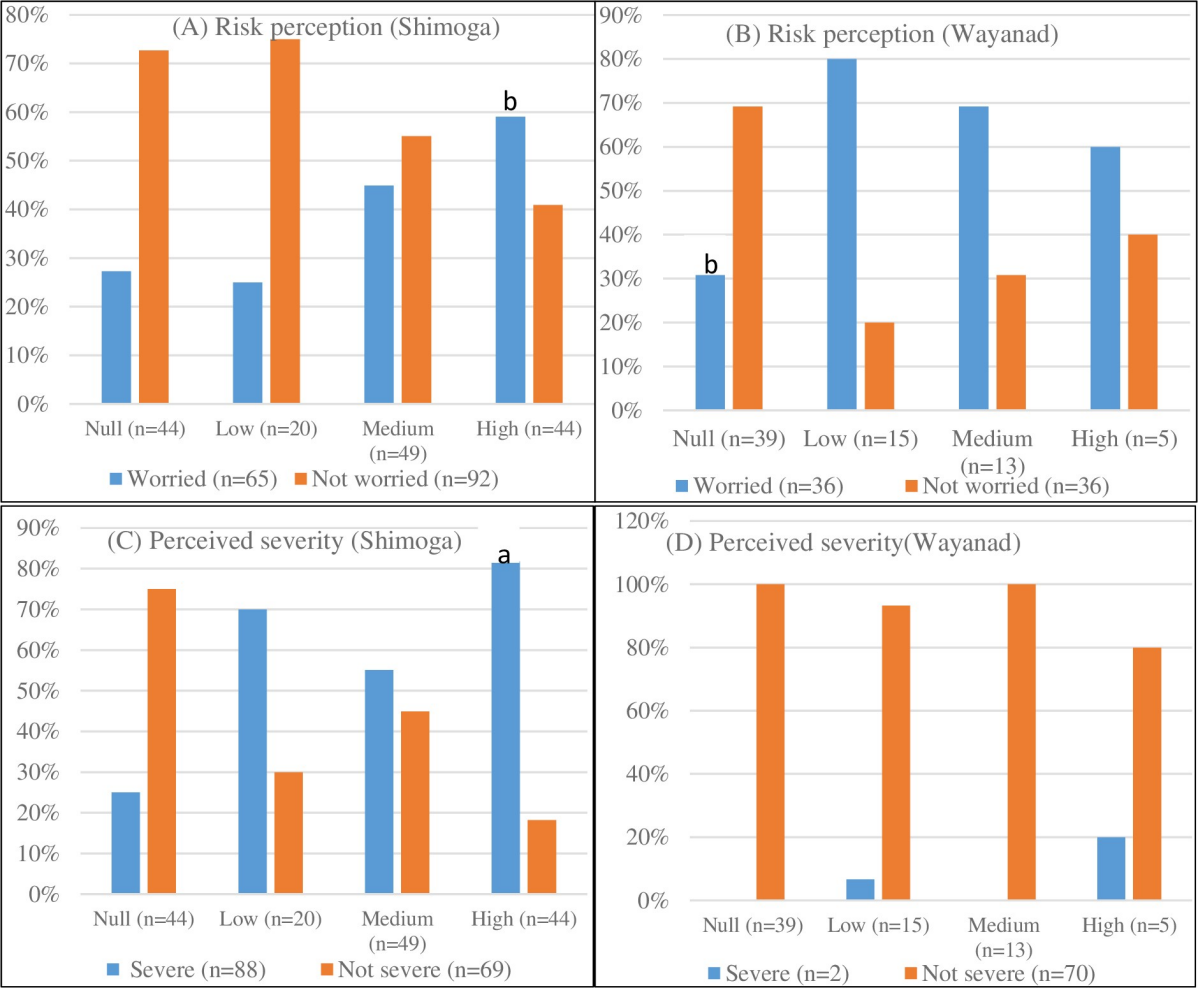
**Cross-tabulation of level of awareness and risk perception (A) worried about contracting KFD and level of awareness [Shimoga]; (B) worried about contracting KFD and level of awareness [Wayanad], (C) Perceived severity and level of awareness [Shimoga], (D) Perceived severity and level of awareness [Wayanad].** ^a^ Significant at p ≤ 0.01; ^b^ Significant at p ≤ 0.05.

Although the overall results suggest that the majority of households had limited or no awareness about KFD (51.3%), this was particularly telling in Wayanad, with 54.2% (39/72) compared to 28% (44/157) who expressed no awareness about KFD in Shimoga, which is significantly lower (χ^2^ value 14.60, p<0.05). Likewise, the proportion of households which reported high awareness of KFD was significantly higher in Shimoga (28.0%, 44/157) as against 6.9% (5/72) in Wayanad (χ^2^ value 13.04, p≤ 0.05). A further cross-tabulation of level of awareness and risk perception (see Fig 3) showed that as many households who expressed high awareness about KFD in Shimoga (59.1%, 26/44) also reported they were worried about contracting the disease (χ^2^ value 7.89, p≤0.05). Similarly, the Wayanad data suggest a far lesser proportion of households who had no awareness about KFD (30.8%, 12/39) equally expressed worry about contracting it (χ^2^ value 14.60, p<0.05). The disaggregated results further indicate that a greater percentage of households expressing high awareness about KFD in Shimoga (81.8%, 36/44) also perceived the disease as severe (χ^2^ value 16.48, p≤ 0.01). A reverse pattern is portrayed in the Wayanad data as the percentage of households that expressed high awareness about KFD (80%, 4/5) also described the disease as not severe. This result was not statistically significant. Altogether, the above results indicate that awareness of KFD is likely to be an important driver of households’ perceived susceptibility to the disease and adaptive decision-making (see Section 4.5).

### Dynamics of access and use of disease information services

To the extent that different disease information pathways have differing implications for local adaptive capacity, it was instructive to distinguish between access to formal and informal information as identified from the empirical data. Formal information refers to the set of information and messages relevant for the prevention and control of KFD generated and/ or disseminated via official institutions or pathways. Conversely, informal information refers to the all the related information and messages received through unofficial channels, including community leaders. This section elucidates and compares these two disease information pathways in terms of the specific channels of dissemination used by respondents and the contents of received information across the two study areas.

#### Formal disease information pathways

**Fig 4:**
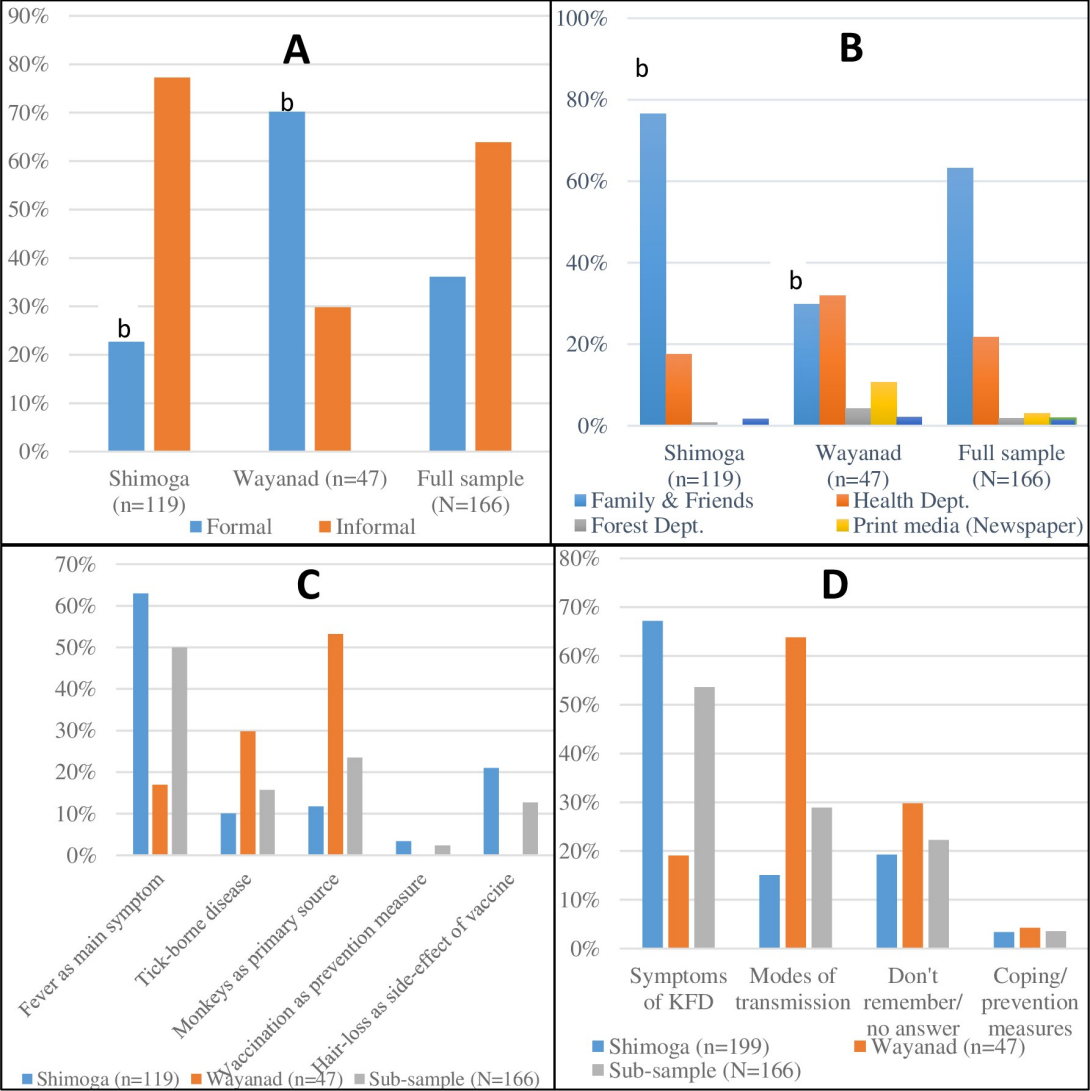
**General sources and typology of received disease information (A) KFD information pathways; (B) specific sources of disease advice, (C) contents of disease information received and (D) typology of disease advice received.** Note: multiple responses. ^a^ Significant at p≤0.01; ^b^ Significant at p≤0.05.

Fig 4 typifies the main modes by which survey respondents accessed disease information in the studied communities. As regards sources of disease information, the overall results indicate important regional differences in terms of disease information pathways. Slightly over two-thirds of households in Wayanad (70.2%) received disease advice via formal sources relative to just 22.7% of households in Shimoga falling within the same category. On the specific formal access modes, the results showed the district health department as the primary source of disease information in Shimoga (17.6%) and Wayanad (31.9%) respectively. Other important sources particularly identified in Wayanad were the Forest Department (4.3%) and the print media (10.6%). Altogether, the overall results appear to give the indication that whereas most households in Wayanad relied on formal sources for their disease advice, corresponding households in Shimoga relied on such sources to a far lesser extent (χ^2^ value 32.97, p<0.05).The survey results was corroborated by the interviews as some participants explained that the regional difference was attributable to the geographical remoteness of the surveyed villages away from healthcare infrastructure and formal disease information services, particularly in Shimoga. Besides, it was also highlighted during the interviews that the limited capacity of local health systems has meant that existing personnel are overstretched and unable to support frequent extension visits or awareness campaigns, especially outside of outbreak seasons in the affected communities. The reflections of a medical officer and taluka health officer during separate interviews in Shimoga are illustrative:

*“Sir*, *problem is distance between houses*, *which is [about] 1 kilometre*. *See*, *how many houses we can visit in a day sir*? *We tell them to do whole village survey in a day but who is there to do*, *junior health assistant will be there and for 1000 population one ASHA (community health worker) will be there*, *in Y village population is 5k*, *but only one ASHA [worker]*, *she can’t even do at least 5 days in a day*.*”* (DM-2, Shimoga)

*“Actually*, *if you look at our department*, *we have to do preventive work and curative work also*. *We are under-staffed*, *so*, *for preventive itself*, *there should be separate staff*. *If I have to do OPD (outpatient care) in the morning and go to field in the noon*, *people will be there in the hospital*, *so there should be separate doctor for preventive aspects*.*”* (TO-2, Shimoga)

To further understand the scope and content of information received (as a function of the quality of disease information), we asked the proportion of survey respondents who had prior information on KFD about two or three key messages they received as shown in Fig 4. The overall results depict an inconsistent pattern in terms of the type and specifics of disease advice received. Whereas the majority of households in Shimoga related to information on the symptoms of KFD (67.2%), households in Wayanad (63.8%) commonly reported information relating to the modes of transmission. This notwithstanding, the overall results in Fig 4C and 4D highlight two important points. First, the contrasting perspectives regarding the role of monkeys as the primary cause of KFD is quite interesting and seems to underscore the limited understanding the disease transmission pathways critical to their adaptation planning (see sub-Section 4.2.2). Second, the overall results surprisingly demonstrate a huge deficit in received information pertaining to coping and personal preventive measures as just 3.6% of the sub-sample (n = 166) reported receiving such information (Fig 4D). Juxtaposing with Section 4.2 (Table 2), the results further reinforces the argument that households generally have limited access to credible information about KFD, particularly on coping and preventative measures. This is particularly telling as over half of the survey sample (51.3% or 118) reported limited or no awareness about KFD, despite the general acknowledgement of the disease as a significant threat to their livelihoods, health and well-being (Table 2). These results highlight a strong need to better contextualise and disseminate comprehensive risk communication messages as it is not access to information per se that affects adaptation, but the mode, scope and quality of information received.

Moreover, the qualitative interviews also revealed some underlying tensions and in a few instances, a sense of distrust between some locals and government-affiliated information brokers, with the former suspecting potential misinformation or bias in the latter’s disease risk communication. There were rumours that some forestry officials deliberately distorted aspects of risk communication by associating KFD exposure with forest usage by communities. Amid the widespread scepticism, some interviewees believed that the KFD-forest usage nexus reinforced a longstanding covert ‘agenda’ of keeping villagers out of the forest. As one forest-watcher in Shimoga, queried the so-called theory ‘tick theory’:

“*I don’t know*, *and I don’t think tick bites will lead to this disease as we had experience with tick bites from the childhood*. *Even this year also*. *I got a good number of tick bites* [he showed the interviewer his legs to ostensibly prove it] *and still now am okay with my health…*” (WD-5, Wayanad)

Indeed, a number of participants (belonging to the tribal groups) acknowledged their underlying ‘place-based’ vulnerabilities (as their hamlets are situated in close proximity to forests and agricultural plantations) to KFD, but expressed strong reservations about the so-called ‘tick theory’ by the Forest Department as a pretext to justify forest access restrictions. A 50-year old tribal woman and a tribal leader in Wayanad had this to say:

*“We are extremely worried about KFD because we have to deal with the forest for income*, *and monkeys are common here*, *but forest officers are restricting entry into the forest and denying us access to forest products*. *When the monkeys are coming to our home*, *stealing vegetables and other things from our garden and kitchen*, *why are these restrictions applied*?*”* (WD-3, Wayanad)

*“Never*, *tick population density does not have any connection with the forest or its usage*. *But you should understand that there is a very clear connection between deforestation*, *plantation and ticks*. *I don’t think that we*, *the Kattunayakan exploit the forest and the forest products*, *we never had a monopoly over this… So the general attitude/ assumption that forest use causes the disease [KFD] is absolute nonsense*. *Every disease may have its own reason for an outbreak*. *But this kind of general assumption and judgment make other people feel about us as virus host*.*”* (WD-1, Wayanad)

#### Informal disease information pathways

Given the remoteness of the surveyed communities to local healthcare infrastructure and formal disease information services, it was pertinent to investigate other (lay knowledge) sources of information were available to households. We therefore asked questions that explored other informal avenues of disease advice such as family members, friends, tribal leaders and traditional healers (as shown in Fig 4). As discernible from Fig 4, a significant proportion of surveyed households (76.5%) in Shimoga accessed relevant disease information mainly through family and friends as opposed to only 29.8% of corresponding households in Wayanad who did so via the same source (χ^2^ value 38.90, p≤ 0.05). Juxtaposing with sub-Section 4.2.1, it is evident from the survey and interview data that most households in Shimoga utilised disease information received from official institutions to a much lesser extent compared with informal sources of information (including traditional/ indigenous knowledge) from within their respective villages such as other family members and friends. This survey finding is significantly supported by the interview data as almost all participants related how valuable informal information exchange between neighbouring farmers, familial groups and other local networks was central in the acquisition of relevant disease information. As a forest-watcher reflected in an interview, neighbouring households often received advice on various social and health-related concerns (including KFD-related information) from perceived local experts which they in turn disseminated to others within their social circles (WD-6, Wayanad).

The widespread recognition of the usefulness of such informal information channels in shaping local KFD knowledge was also countered by a few divergent views highlighting some potential skewness and/ or imperfections in the disease information delivery. Indeed, while conceding that local information brokers (including village chiefs, forest watchers and tribal promoters) constituted valuable sources of disease advice (given their privileged access to different kinds of information from both government and non-government sources), some interviewees implicitly referred to instances where disease information was either inadvertently misrepresented or deliberately ‘withheld’ and only shared amongst pockets of ‘closed’ network of local elites and groups, particularly those belonging to the ‘upper class’ groups. For instance, one female ASHA worker in Wayanad in emphasising the importance of a better understanding of the disease in their risk communication recounted instances where some information brokers inadvertently misrepresented KFD as primarily caused by monkeys (as the name *monkey fever* appear to suggest) instead of the KFDV transmission by infected ticks due to limited knowledge about the disease. As she further remarked:

*“I never knew that these small ticks harm people like this*. *I think most people get confused with the disease name even though monkeys do not have any role in it*. *We should give more priority to understanding the disease*. *Otherwise*, *it always misleads [even] the ‘literate’ people*. *Then just imagine how ‘illiterate’ people understand this disease*. *Most of the news [about KFD] passed through inter-personal conversations*, *which again makes them [villagers] feel that monkeys are their enemies as monkeys spread the disease*.*”* (WD-2, Wayanad)

*“…Now*, *the monkey has been brought [by the forest officers] from outside*, *those monkeys will not be in the forest*. *Those will be around the home*. *That is a problem*. *Monkeys used to stay in the forest and was not in the habit of coming to the villages*. *Monkeys which are brought from the city*, *will not go to the forest*, *they will be around the houses*. *That is the main problem*. *Foresters unload the monkeys here*, *many people have has seen two to three vehicles [bring them in]*. *Everything started 15 to 20 days later after the unload…”* (SA-4, Shimoga)

A number of participants further disclosed that they changed their perceptions and made new decisions after information exchange with their neighbours and friends pertaining to KFD. Within this context, the interview data further revealed that participants who had prior access to KFD-related information from formal and informal sources seem to demonstrate a clear adaptive advantage relative to those with limited or no access to disease information. The respective testaments of a tribal promoter and a medical officer in Wayanad and Shimoga during separate interviews are illustrative:

*“We are aware about this disease from A to Z*, *even the life cycle of ticks*, *which seasons they are abundant*, *and what preventive measures are required*. *Even though we are not practising all of them…*, *we reduced number of days* [*traditionally spent two weeks*] *we live in the forest in search of honey and started wearing footwear and other natural materials to protect ourselves against tick bites…”(*WD-1, Wayanad)

*“We have a separate dress for work and a separate dress for home use*, *since previous days*. *I wash the dress on alternative days*. *We usually use tick prevention medicine for cattle once in a month or 5 months*. *We wash [cattle] with singe powder*. *We do bath daily*, *use oil for the body*.*”* (SA-2, Shimoga)

These statements highlight the view that existing social networks shape the scope and quality of disease information received to a large extent. Yet, access to critical disease information via such informal brokers appeared to be somewhat differentiated along the lines of socio-cultural affiliations. A case in point is the story of a 52-year old widow belonging to a scheduled tribe in Wayanad who lamented the demise of her husband partly due to lack of prior awareness about KFD:

*“When my husband passed away due to this disease that was the first time I heard of this monkey disease*. *Even when he was admitted in the hospital no one* [*in my village*] *told me about this disease*. *But when he died*, *people told me about the disease… He was engaged with some normal activities only*, *every year he went for honey collection*, *then medicinal plant collection and grazing…So I can’t explain how he got it* [sobbing].*”* (WD-3, Wayanad)

### Adaptive practices and utilisation of disease information

**Fig 5:**
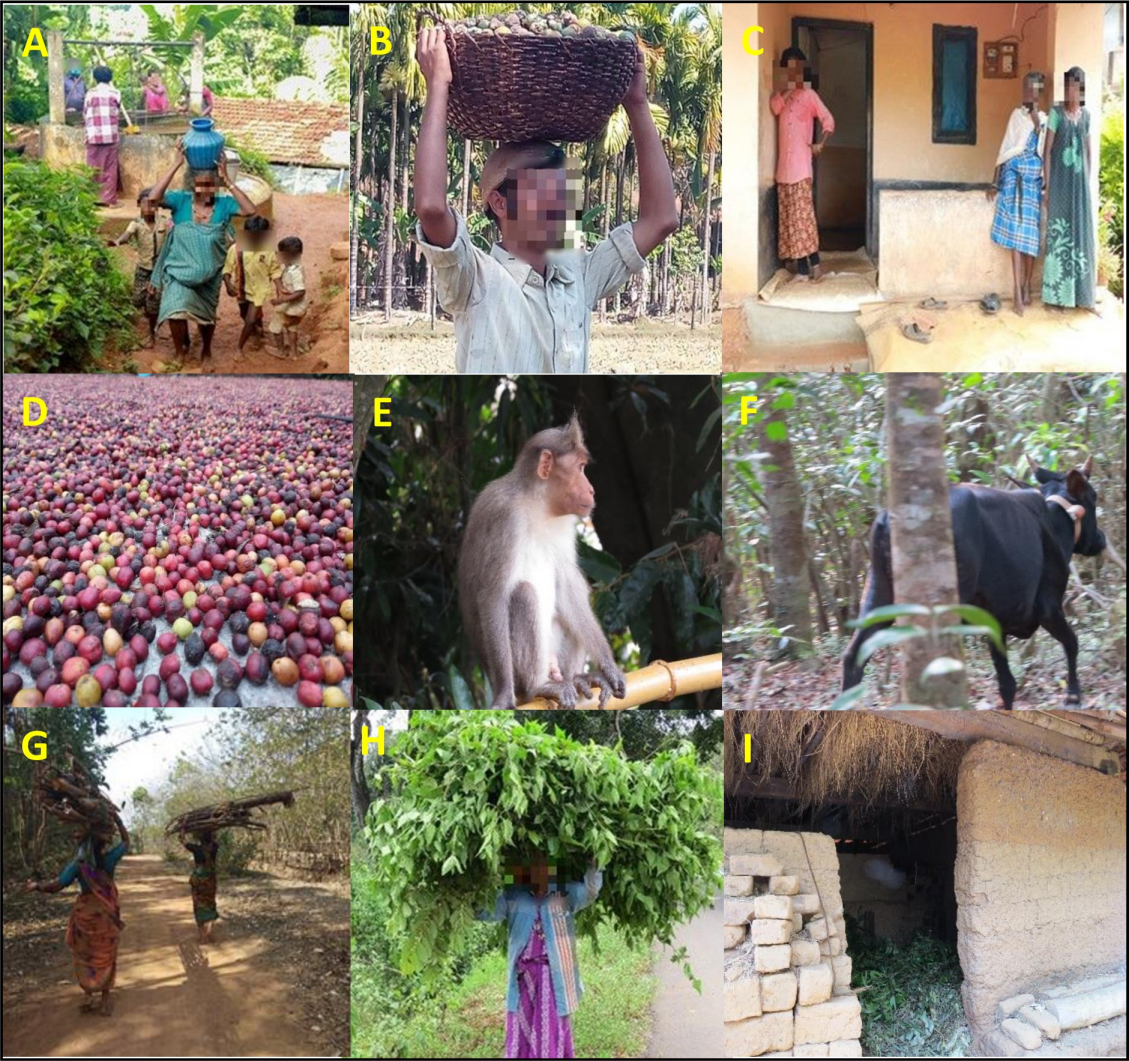
A household livelihood matrix depicting aspects of human-environment interactions in a village in Wayanad. **(A) A middle-aged ‘tribal’ woman carrying a pot of water and her children walking bare-footed; (B) farmer carrying basket full of harvested arecanut seeds from a nearby field; (C) a lady wearing a long sleeve [men’s] shirt and trousers as she prepares to go to the forest for firewood collection, and two other ladies wearing their traditional dress and it is visible that they stated using foot-wears following the outbreak; (D) harvested coffee seeds; (E) an example pale-bellied bonnet macaques (*macaca radiata diluta*) implicated by locals for causing KFD; (F) a cow openly grazing in a nearby forest patch; (G) woman carrying firewood; (H) woman carrying harvested leaves from nearby forest; (I) cow shed with leaf litter.** Photo credit: Mujeeb Rahman, Darshan Narayan & Stefanie Schafer.

**Fig 6:**
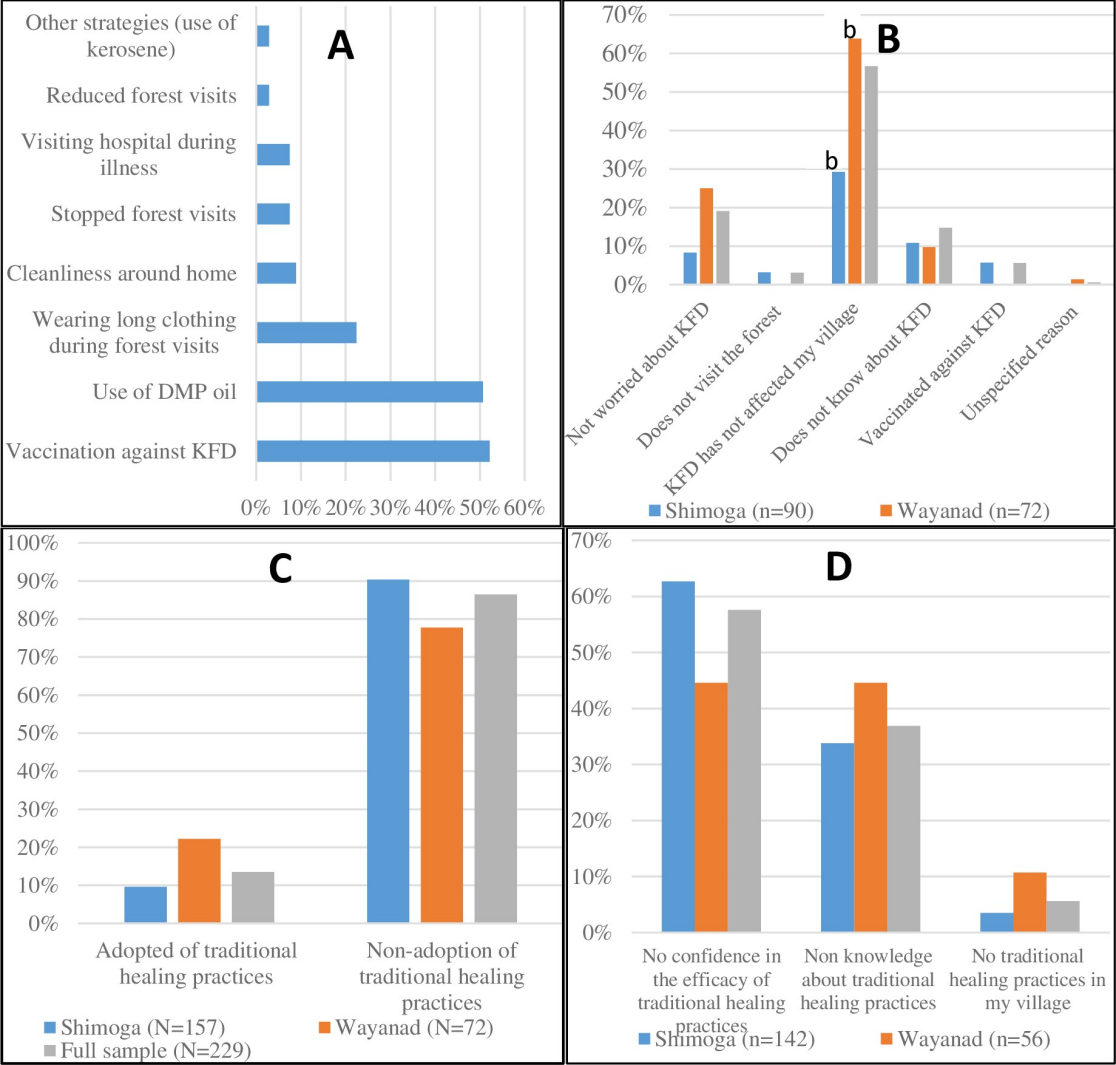
**Changes to household lifestyles due to KFD (A) Specific adaptive strategies employed based on Shimoga data; (B) Reasons for not altering lifestyle, (C) Adoption of traditional healing practices,** (D) Reasons for non-adoption of traditional healing practices. ^a^ Significant at p≤0.01; ^b^ Significant at p≤0.05.

Given that disease information is critical to reducing vulnerability of households and bolstering their adaptive capacity to emerging disease risks, it was instructive to explore further how the utilisation of disease information contributed to households’ adaptive capacity in the face of the recent KFD outbreaks in the surveyed communities was further explored. In this context, respondents were first asked whether or not their respective households modified their lifestyles in response to the potential impacts of KFD (see Fig 5, for a snapshot of the day-to-day people–environment interactions captured in a livelihood matrix). In Shimoga, whereas 42.7% of respondents affirmed that their respective households did change their lifestyles as part of their adaptation planning, 57.3% indicated otherwise. By contrast, the Wayanad data showed that none of the surveyed households altered their lifestyles in response to the potential impacts of KFD (Fig 6). Altogether, the overall results highlight important regional differences in adaptation planning, with the majority of households (representing 60.7%) not altering their lifestyles in response to KFD and its associated impacts.

Regarding the specific coping strategies’ employed to safeguard against exposure to KFD, households that confirmed altering their lifestyles, particularly in Shimoga, were further asked about the specific ways by which they did so. As illustrated in Fig 6A, the results show multiple coping measures employed by individual households, with vaccination uptake (52.2%), use of DMP oil (50.7%) and wearing of long clothing during visits to the forest (22.4%) as the foremost coping strategies employed. As to why nearly two-thirds of the total sample (60.7%) did not implement any adaptive measures, three common reasons were adduced by survey respondents, including the fact that KFD had not yet affected their village (56.7%), respondents were not worried about contracting KFD (19.1%) and had limited awareness about KFD (14.8%). Further disaggregation by study area revealed marked regional differences in responses relating to the absence of KFD in some villages, as at the time of the survey, as the primary reason for inaction. For instance, the Wayanad data indicates that households that cited the absence of KFD in their villages as the key reason for their inaction constituted more than double (63.9%) the proportion of surveyed households in Shimoga (29.3%) reporting same (χ^2^ value 24.57, p≤ 0.01). The survey results are corroborated by the interviews as most participants expressed the lack of prior knowledge and/ or awareness of the disease emergence in their village as the underlying reason for the limited implementation of adaptive measures.

To address the question of how the utilisation of disease information contributed to households’ adaptive capacity, a further cross-tabulation analysis was undertaken. When disaggregated by study area, the Shimoga data showed no significant association between access to disease information and alteration of lifestyles, as the majority of households who had awareness of KFD did not modify their lifestyles as the result of the disease emergence. At face-value, these results seem to rather puzzlingly contradict the a priori assumption that access to disease information is an essential prerequisite for adaptation planning. Yet, further cross-tabulation analysis revealed that as many households that had prior experience of KFD in Shimoga (68.4%, 26/38) also reported altering their lifestyles following the disease emergence (χ^2^ value 10.72, p≤ 0.05). This gives the indication that prior experience rather than awareness of disease risk per se influences adaptive behaviour. As our interview data confirms, the overall picture of adaptive decision-making is more nuanced and complex. Indeed, most participants expressed limited belief in their own ability (agency) to change their circumstance, which invariably constrained the translation of received disease information into substantive adaptive action. For instance, a number of interviewees in response to the question of how they adapted to KFD worriedly remarked “*we believe in God to protect us*”, suggesting that they were resigned to their fate in the face of a looming disease risk. While this finding may in some sense cursorily connote limited opportunities for utilising disease information to inform substantive adaptive actions, critically however, it implicitly echoes the view that individuals and households’ understanding and realities of KFD are socially and culturally constructed. To the extent that this inference holds true, then it also conveys the understanding that the lived realities and perceptions of local communities about (zoonotic) disease risks cannot be divorced from underlying socio-cultural beliefs and practices [33,34]. Moreover, the limited agency of households could be indicative of other pre-existing structural inequalities which operated to constrain their capacity to act on received disease advice in practice. In fact, a recurrent theme from the interviews was that aside from the seasonal impacts of KFD, other socio-economic inequalities and issues not only limited their ability to cope but significantly exacerbated the impact of the disease stress. Two typical views in this regard were given by a PHC medical officer stationed in Shimoga and tribal leader in Wayanad:

*“See to be honest*, *the number of deaths that occurred here did not happen anywhere else*. *Why were there more deaths*? *If we were to contemplate on this*, *especially in this village*, *it would be due to many factors*. *Low socio-economic status*, *poor nutrition*, *and poor immunity… Talking about the minimum lethal dose of virus*, *due to the high tick positivity density*, *tick bites are higher*, *and the viral load is higher*. *That’s why the deaths are higher*.” (DM-2, Shimoga)

*“Elderly people [are] more vulnerable as the intake of alcohol in good quantity and malnourished women comes second as most of them are not eat proper food properly*. *Even we are getting subsidised food grain for free but we are unable to improve their condition…If you see the average age of death case*, *you can actually understand my points*.*”*(WD-1, Wayanad)

### Disease adaptation in practice–looking beyond the ‘techno-managerial’ approach to KFD vaccination

The lack of specific treatment vis-à-vis the seasonality of KFD outbreaks (November to May) has meant that vaccination remains the single-most important adaptive strategy to the disease [22,28,29]. Yet, in Table 2, the overall results suggest a limited uptake of vaccination (46.7%), despite nearly two-thirds of the sample (72.5% or 166) having had prior information about KFD (χ^2^ value 4.865, p≤0.05). Although consistent with the literature and thus not surprising, it is particularly telling that of the proportion of households who had prior information about KFD in Wayanad (65.3%), only 19.4% were reportedly vaccinated against KFD (χ^2^ value 9.244, p≤0.05). Further cross-tabulation analysis revealed statistically significant differences between socio-economic characteristics and vaccination, with uptake strongly influenced by caste affiliation, poverty, prior experience of KFD, and awareness of KFD transmission pathways. For instance, the disaggregated results indicate that lower-caste households reported higher vaccination uptake than upper-caste households (χ^2^ value 15.569, p ≤ 0.05). Likewise, a higher percentage of BPL households relative to APL households reported uptake of vaccination against KFD (χ^2^ value 15.100, p ≤ 0.05). Further disaggregation by awareness of KFD transmission by infected tick-bites and repeating the above quantitative comparisons, we found that a far lesser proportion of households who did not perceive KFD as severe claimed to be vaccinated as against household indicating otherwise. Moreover, the overall results also indicate significant differences between prior experience of KFD and vaccination status. For instance, a higher percentage of households that reported prior experience of KFD in Shimoga (78.9%, 30/38) also claimed being vaccinated against it (χ^2^ value 6.34, p ≤ 0.05). A similar picture is depicted in the Wayanad data with the majority of households with no prior experience of KFD reported not being vaccinated against it (χ^2^ value 6.13, p ≤ 0.05).

**Fig 7:**
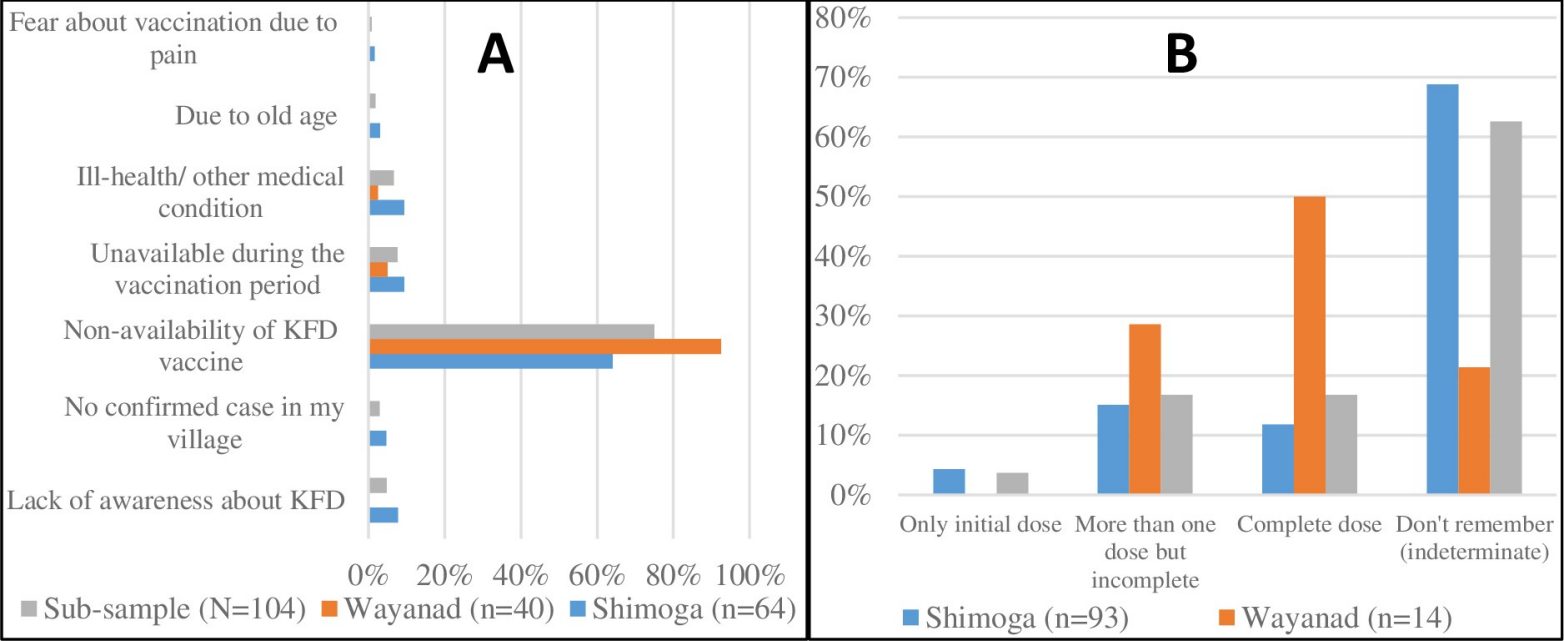
Household vaccination against KFD (A) Reasons for not taking vaccination against KFD; (B) Number vaccine doses taken.

These results (which are also supported by our interview data) seems to put to question the simplistic stereotypical view that marginalised populations are less receptive to vaccination against KFD. Nuancing this narrative, they convey the understanding that the supposed hesitance or reticence of vulnerable groups to KFD vaccination could well be attributable to the limited access to quality disease information and to some extent, the often-overlooked but deep-seated [trust] concerns about the efficacy current vaccine (see Fig 7). The fact that a higher percentage of households that expressed awareness of KFD transmission by infected tick-bites (45.8%) as against households stating otherwise (1.9%) equally reported vaccination uptake, further buttresses this observation (χ^2^ value 15.100, p≤ 0.01). Indeed, participants during the interviews flagged a myriad of concerns about the existing vaccine (including lack of knowledge, required multiple-doses, inappropriate timing, and pain) which hampered widespread uptake of vaccination, especially in Wayanad. Reflecting the need for improvement in the existing vaccine, a male KFD survivor (plantation worker) in Shimoga said:

*“This time nothing is done*, *why you know there was a positive case this time but they had taken booster dose*. *This means vaccination*, *not effective I feel*. *It is useless…Health department people advice*, *they really tried well but we don’t know why it* [KFD] *comes*?*”* (SA-3, Shimoga)

These sentiments were also widely shared by the disease managers as well:

*“Only problem is with*, *in my view*, *the vaccine…vaccine is the main hitches*. *Because the acceptance of that vaccine is not so [great]…welcoming sign is not seen*. *One more point is the doses also*. *We need to give multiple doses to get what we need*. *To get some protection he/she needs to take full course*. *After that every year he/she needs to get booster dose for five years*. *Those are all hitches*. *I think one single injection that can protect the person for five years is needed…*.*”* (DO-3, Shimoga)

*“…We are struggling with the age-old vaccine*, *which was prepared in the 90s I think*. *We are going with the same*. *We don’t know about the strain change…the virus…Even the research has not* [been] *done*. *Even the cases which (who were) vaccinated fully*, *also were [re]infected*. *So*, *for that we need to do some research on whether the prevalence has changed it or not*.*”*(DO-4, Shimoga)

Another important barrier to households’ uptake of vaccination relates to the existing religio-cultural beliefs and practices. It was gathered from the interviews that some participants particularly those of scheduled tribe or caste backgrounds strongly perceived vaccination as something ‘alien’ from their long-standing religio-cultural belief systems. A tribal leader from Wayanad was quite critical of about the ‘techno-administrative’ approach to KFD vaccination highlighting that “*I am totally against the vaccination strategy of the government*, *until I get clear knowledge how the vaccination works in our body to prevent the KFD and both advantage and disadvantage of taking vaccine…”* (WD-4, Wayanad). Corroborating this stance was another tribal promoter from *Kattunayakan* village (in Wayanad), reflecting on his own experiences with KFD vaccination, posited that despite the government’s free supply of protein-contented food (including milk, boiled eggs etc.) as way of incentivising vaccine uptake, some tribal groups were still somewhat reticent suggesting *“it was not in our blood*, *mostly people who are above 40 never taken any vaccine*”. The tribal promoter further remarked:

*“We [Kattunayakan] are not that much literate and we speak another dialect*, *and most of the young people can follow the Malayalam language very easily but the people above 45 will not understand what people speaks about*. *So it was me who convinced the people and myself I was struggling to understand how serious it [KFD] was*. *And it took almost a year to understand the risk of KFD*, *meanwhile we lost more than 16 people*.*”* (WD-1, Wayanad)

Other important contextual factors that impeded widespread vaccination uptake that emerged from the interviews were the limited health extension and inaccessibility of some villages. Altogether this conveys the understanding that maintenance of social relations and networks were important avenues for receiving disease information, which in turn shaped the ability to adapt. This is consistent with the observation that lived experiences and perceptions cannot be divorced from their socio-cultural beliefs. It therefore follows that trust building and concerted community sensitization remain important to obtain the requisite buy-in and demystify otherwise deep-seated (anti)vaccination perceptions.

Aside from vaccination, livelihood diversification emerged as another important coping strategy adopted by households following the KFD outbreak. A number of participants narrated how they have had to temporally switch from the collection of non-timber forest products (NTFPs) such as honey and firewood to wage labour. Even so, such adaptive action was contingent on the availability of such non-forest based jobs and some had to care for ailing relatives, implying they had limited time to effectively engage in alternative work. Whereas government intervention through (alternative) employment and social welfare schemes have in some respects afforded a potential leeway to adapt, thus alleviating the otherwise grave impacts of the outbreak, it was also gathered that the implementation of these support schemes had inadvertently contributed to other unanticipated maladaptation consequences. According to another *Kattunayakan* tribal leader, whereas some households managed to secure temporary employment as construction labourers (earning on average Rs. 900 [£9.60] per day), this coping strategy nonetheless occasioned and/or exacerbated other existing communal problems, including ‘adulteration’ of traditional value systems, ‘politicisation’ of tribal women, and alcoholism amongst others:

*“The one which I felt very personally*, *that is government intervention through the employment schemes and its implementation is taking this community away from how they lived so far*. *I am not blaming the government*, *neither the employment scheme*, *but unfortunately as we are tribe*, *we should have something unique (that we going to lose)*. *Our name then (kurmar) is going to be lost*. *As the disease spreads*, *the government brings more restrictions and forest use will become impossible*. *You can call it an identity crisis of the tribe*, *but I am sure*, *the coming generation will never follow the rituals as we practice now…”* (WD-1, Wayanad)

## Discussion

Small-holder and tribal communities face a plethora of challenges including exposure to zoonotic disease risks that significantly constrain their livelihood opportunities and general wellbeing in LMICs, including India [4,2]. Within this purview, there is a growing consensus in the ‘One Health’ literature that reducing vulnerability to endemic and emerging zoonotic diseases is heavily predicated on reducing poverty and existing socio-economic inequalities that impede the individual and collective agency of vulnerable groups [30,31,13]. This study thus sought to understand how the dynamics of access to and utilisation of disease information services shaped the adaptive capacity of small-holder and tribal communities, which constitute the most vulnerable of the vulnerable groups, drawing on two case studies in the Western Ghats region of southern India.

Whereas our findings broadly suggest that access to and utilisation of disease information has the propensity to improve households’ adaptive capacity against KFD and its associated stressors, the ability to leverage and act on such received information was somewhat dampened by pre-existing socio-cultural and economic inequalities, particularly caste and poverty status. Key marginalised groups such as tribal women, lower caste households, etc. seem to exhibit potentially weak adaptive capacity as did those from BPL (below poverty line) backgrounds. Even though the majority of households did not express worry about contracting KFD per se, this is not indicative of indifference but rather echoes an underlying sentiment of helplessness in the face of adversity as most participants indicated they were perturbed by the potential impact on their livelihoods and welfare, including loss of income, loved ones, caring for ailing relatives and limited access to medical care. Indeed, some tribal households characterised KFD as an existential threat expressing concerns that it could potentially lead to an ‘identity crisis’, redefining their longstanding traditional social and livelihood patterns. As evidenced in sub-Section 4.2.1 majority of households perceived KFD as a significant health concern in their respective communities. The foregoing observation reinforces Bardosh et al.’s [13] argument that different group of people respond differently to disease interventions owing to differences in their socio-economic capacities. Besides, it also highlights the limits of existing techno-administrative interventions which somewhat lends empirical credence to Power et al.’s [51] argument that given limited agency to adapt people may adopt a positive identity or ‘hopeful adaptation’ in the face of adversity, which perhaps suggests a strong (mal)adaptation effect.

Consistent with observations in the literature, the overall results suggest low uptake of vaccination (46.7%) and other personal protection measures (such as usage of tick repellents and wearing long-sleeve clothing during forest visits) which significantly constrained disease adaptation [22,28,29]. While not surprising, this result is telling considering that nearly three-quarters of the sample (72.5%) had reportedly received information about KFD (see Section 4.5). This supports the view that whereas access to disease information is necessary for vulnerable groups’, it is nevertheless not a sufficient condition to strengthen their adaptive capacity, in terms of their propensity to implement adaptive strategies to sufficiently adapt. Of the variety of information sources cited by households, we found that informal sources were important channels for disseminating KFD-related information. Admittedly, the health department’s information education and communication (IEC) outreach activities were also severely constrained by logistical and personnel challenges, which highlights the need for strengthening technical capacities and more importantly, cross-sectoral collaboration as a means of complementing existing management efforts [52,22,16]. There is some evidence that tailoring risk communication with potential adaptation pathways (including leveraging relevant traditional practices) and presenting these in relatable and locally appropriate languages could potentially incentivise widespread uptake of disease information [16].

Importantly, aside from ‘technical’ information that is formally received, households also resorted to other informal channels for urgent disease information, including KFD. Indeed, about 76.65% received virtually all their disease information or knowledge from ‘family and friends’ (see sub-Section 4.3.2). Yet, the importance of these (lay) sources of knowledge in the arena of (zoonotic) disease adaptation is still relatively under-explored and strikingly absent in current policy discourses, at least on KFD management in India. Information-sharing within and across networks was also vital in adaptation considerations [10,37]. Yet these risk misrepresentation as evidenced in our results and dissuades others from undertaking certain effective adaptation measures (such as protective measures against ticks). These results suggest that there is an important window of opportunity to further intensify and widen the scope and reach of current disease awareness campaigns through local information brokers such as tribal leaders, religious leaders and traditional healers given their strong legitimacy and trust within their local socio-political settings.

Our results also suggest that adaptation and disease information is socially and culturally constructed, and mediated by social networks, gender and socio-economic status which is broadly consistent with the literature that demonstrates how differential access to productive resources are shaped by prevailing social and political structures at different scales [35,53,13,30]. It therefore follows that addressing vulnerability to KFD and bolstering the adaptive capacity of forest communities hinges critically on addressing poverty and other socio-economic inequalities [10,12]. Considering that the social and livelihood organisation of forest communities are conditioned around ‘secure’ forest access, existing and future interventions on zoonoses management must strongly reflect this reality [1,16]. As evidenced in our results, the seeming scepticism regarding messages on the voluntary avoidance of forest and/ or compliance with imposed forest restrictions (in a bid to reduce the risk of forest communities’ exposure to KFD) reflects the operational limitations of ‘top-down’ and paternalistic approaches of the bureaucracy on the ground. This observation lends credence to the argument that the interplay of opposing stakeholder interests could potentially lead to the distortion of otherwise credible information, thereby exacerbating pre-existing socio-economic exclusions. Conversely, the scepticism of some local groups highlights a significant ‘communication gap’ between policymakers, disease managers and local communities (see Section 4.3.1). This practical dilemma underscores the importance of trust building as foundational in gaining legitimacy of forest communities, especially tribal groups, considering the historical tensions with the Forest Department over forest tenure and access, which seem to have somewhat ‘marred’ interactions with these communities [54,55]. Moreover, the identified knowledge gaps in the ecology and epidemiology of the KFD system such as the precise role of monkeys in KFD transmission, tick densities and distribution within affected landscapes, and the duration and percentage immunity conferred by the existing vaccine further highlight the need for concerted community engagement at multiple levels.

Altogether, our findings underscore the view that context-specific interventions predicated on a better understanding of the mutual conflicts between reducing disease vulnerability and safeguarding livelihoods, as well as putting adaptation planning at the heart of KFD management decision-making remain critical. In the absence of a clear synergy or policy support to connect vulnerable communities to successful long-term adaptation pathways, it is difficult to see how merely providing vulnerable communities with disease information alone will bolster their capacity to effectively implement adaptive measures that safeguards their livelihoods, health and well-being. This certainly calls for creating effective linkages and participatory approaches to coordinate disease knowledge, information-sharing and alternative livelihood options among and between key stakeholders, including research and extension services support.

The study has a number of limitations. The cross-sectional nature of the dataset has meant that important differences that might exist in changing adaptation could not be explored. Another issue perhaps relates to the limited interpretability of findings as they might be subject to self-reported recall bias. Furthermore, whereas the positionality of the interviewers as ‘insiders’ (i.e. natives) facilitated rapport building with participants, that could have also ‘coloured’ their attention to some otherwise promising lines of enquiry as they might already be familiar with some of the observations by the key informants. This observation is premised on the implicit assumption that the research process cannot be completely decoupled or divorced from the researchers’ own socio-cultural orientation and experiences. Thus, the generalisability of the quantitative and qualitative findings to other contexts with varying socioeconomic characteristics and health systems should be done with caution albeit the more abstract findings related to constrained individual and collective agency and its role in shaping adaptive responses could be revisited in other similar settings. Nevertheless, the combination of qualitative and survey data afforded the unique opportunity to sufficiently explain and capture some local-level nuances in households’ disease information access and adaptation planning, which otherwise would have remained unobserved or hidden in a wholly quantitative study.

## Conclusion

This study focussed on access to and utilisation of disease information as a potential determinant of adaptive capacity at the local level using evidence from smallholder and tribal households in two case study districts in southern India. This is against the backdrop of the relative dearth of empirical focus on access to disease information services, particularly related to endemic zoonotic diseases in enhancing the adaptive capacity and resilience of vulnerable groups to growing disease threats in LMICs, including India. The present study inter alia demonstrated that small-holder and tribal households valued disease information as critical to their adaptation to KFD. Of the myriad of sources, respondents concurred on their perceptions and experiences that formal and informal channels of information dissemination both played a role. Yet it was observed that different households had differential capacities to act on received information. In other words, they had limited agency to translate disease information into substantive adaptive actions that bolstered their adaptive capacity to the KFD.

To enhance adaptive capacity and by extension resilience of households to KFD and safeguard livelihood security, it is important for farmers’ local-level knowledge and actions to be augmented by government and other institutional support for effective and successful adaptation. While acknowledging that there are ongoing disease control efforts, we argue that there is a need to improve access to credible and timely information to bolster adaptive capacity of vulnerable groups. This requires adequate and tailored policy and institutional support at the local level. These observation are premised on the understanding that disease adaptation is context-specific and requires effective coordination of religio-cultural and political forces, which suggest that disease information is necessary but not a sufficient condition for successful adaptation. Other important contextual factors operated in tandem with household and extra-household level factors to exacerbate existing inequalities which altogether constrain households’ ability to transform received information to substantive adaptive actions. It therefore stands to reason that broad-based strategies (that leverage opportunities afforded by cross-sector collaboration in terms of wider information dissemination and support networks) are critical for bolstering adaptation for vulnerable groups in the face of emerging disease risks. This invariably requires more empirical evidence to explore the short and long-term impacts of adaptive practices by local stakeholders to better understand the scope and extent of vulnerability within and across groups, especially in forest communities highly dependent on forest-based resources for their livelihoods. This remains vital to inform ongoing and future zoonotic disease interventions in India and elsewhere.

## Supporting information

S1 Table Main thematic analysis results summaries based on key informant interviews with KFD survivors, district and taluka managers regarding their experiences and perceptions about 2018/19 KFD outbreak in the Western Ghats area of India. (TIF)

S2 Table Key informant interviews. (TIF)
