# Supplementary figures and images for: ‘None of my ancestors ever discussed this disease before!’ How disease information shapes adaptive capacity of marginalised rural populations in India
Source: PLoS Negl Trop Dis. 2021 Mar 11;15(3):e0009265. doi: 10.1371/journal.pntd.0009265 (PMC7987196; doi:10.1371/journal.pntd.0009265)

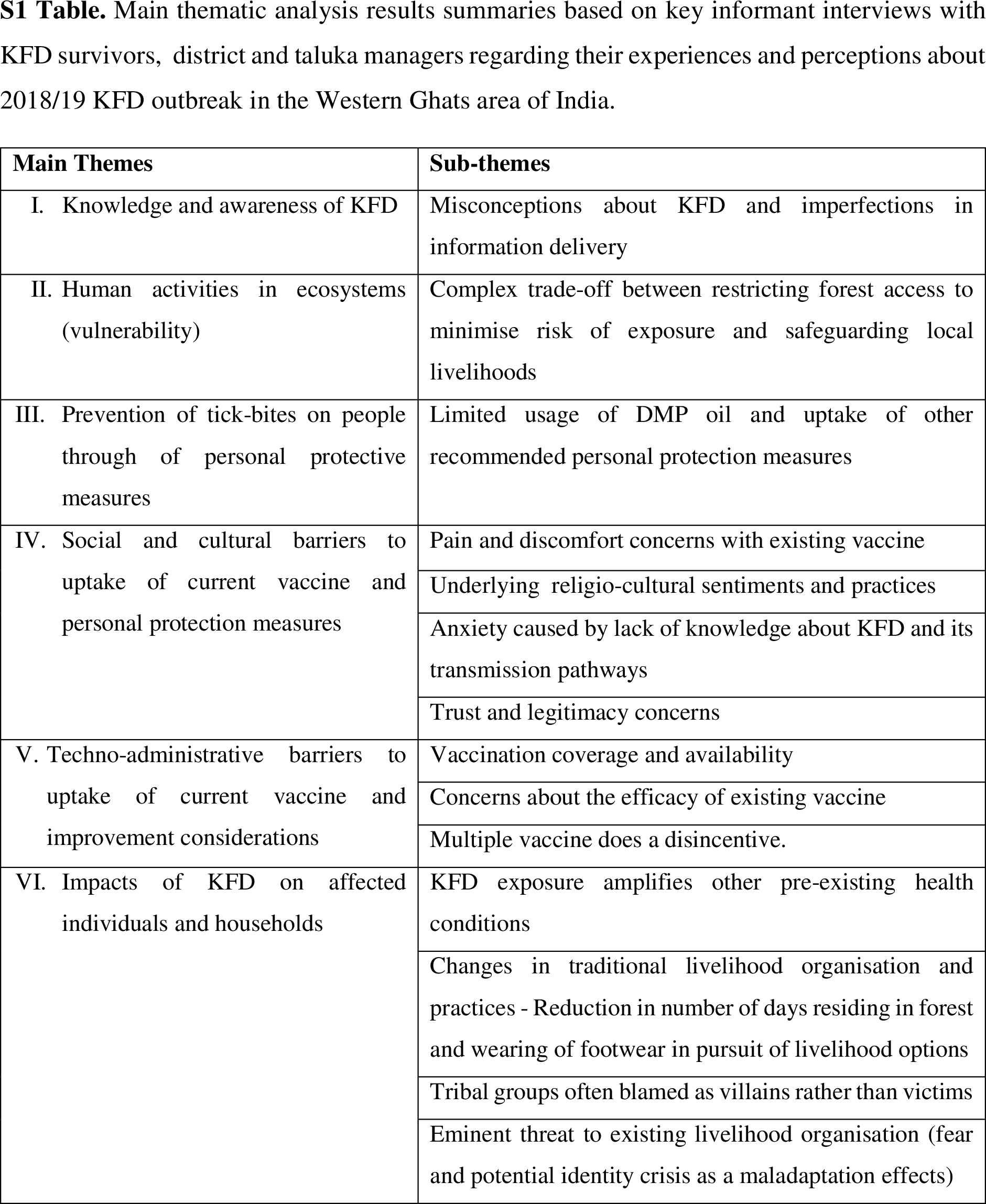

Supplement: S1 Table — (TIF) [file pntd.0009265.s001.tif]

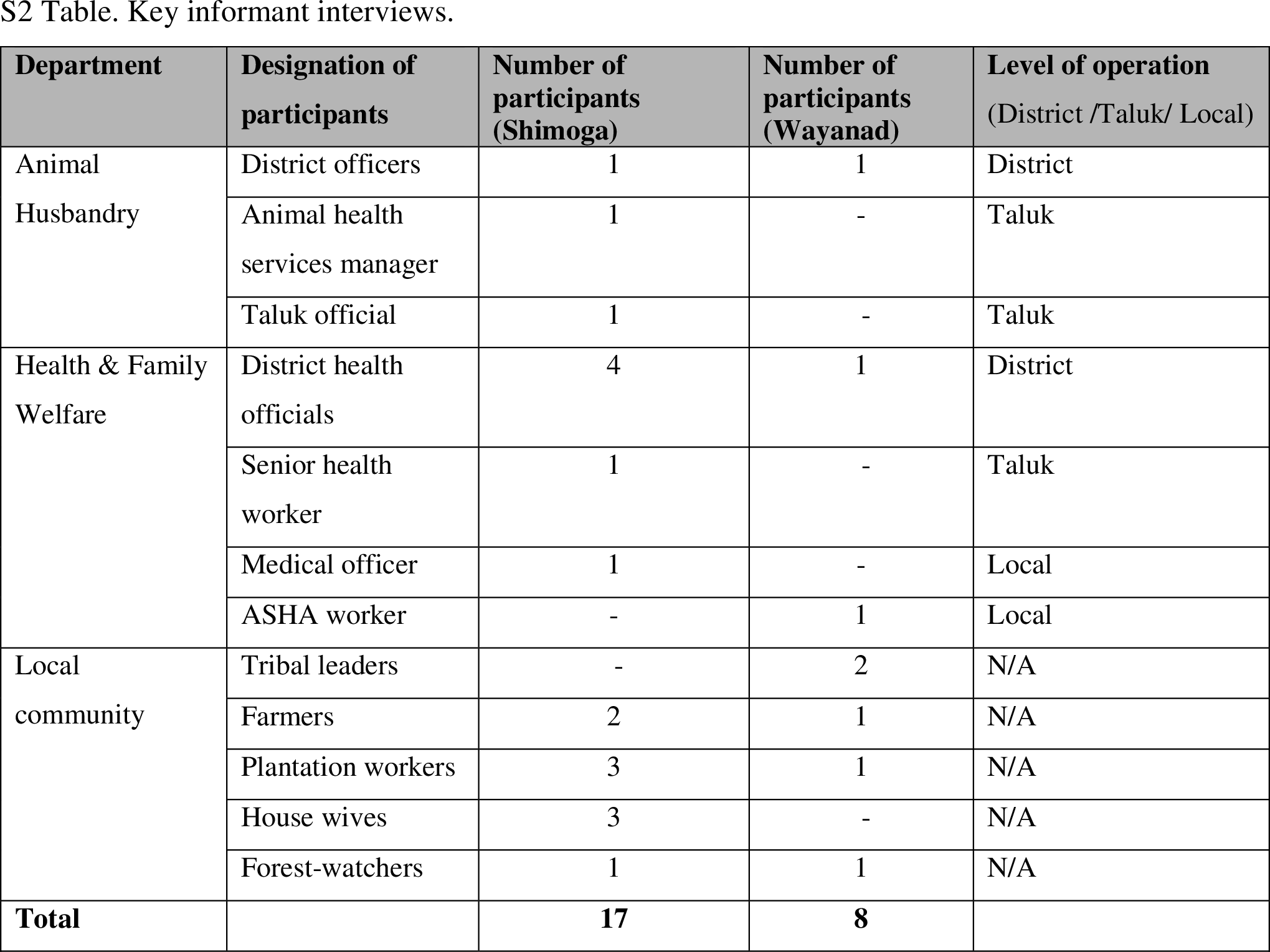

Supplement: S2 Table — (TIF) [file pntd.0009265.s002.tif]
